# Combination tumor‐treating fields treatment for patients with metastatic non‐small cell lung cancer: A cost‐effectiveness analysis

**DOI:** 10.1002/cam4.7070

**Published:** 2024-03-12

**Authors:** Kun Liu, Youwen Zhu, Hong Zhu, Manting Zeng

**Affiliations:** ^1^ Department of Oncology, Xiangya Hospital Central South University Changsha Hunan China; ^2^ National Clinical Research Center for Geriatric Disorders, Xiangya Hospital Central South University Changsha Hunan China

**Keywords:** cost‐effectiveness, docetaxel, immune checkpoint inhibitor, metastatic non‐small cell lung cancer, tumor‐treating field

## Abstract

**Background:**

Tumor‐treating field (TTFields) was a novel antitumor therapy that provided significant survival for previously treated metastatic non‐small cell lung cancer (mNSCLC). The consistency of the cost of the new treatment regimen with its efficacy was the main objective of the study.

**Methods:**

The primary parameters, derived from the Phase 3 LUNAR study, were collected to evaluate the cost and efficacy of TTFields plus standard‐of‐care (SOC) (immune checkpoint inhibitors [ICIs] and docetaxel [DTX]) or SOC in patients with mNSCLC by establishing a three‐state Markov model over a 15‐year time horizon. Primary outcome measures for this study included costs, life‐years (LYs), quality‐adjusted LYs (QALYs), and incremental cost‐effectiveness ratios (ICERs). Sensitivity analyses were performed.

**Results:**

The total costs of TTFields plus SOC, TTFields plus ICI, and TTFields plus DTX were $319,358, $338,688, and $298,477, generating 1.23 QALYs, 1.58 QALYs, and 0.89 QALYs, respectively. The ICERs of TTFields plus SOC versus SOC, TTFields plus ICI versus ICI, and TTFields plus DTX versus DTX were $613,379/QALY, $387,542/QALY, and $1,359,559/QALY, respectively. At willingness‐to‐pay (WTP) thresholds of $150,000/QALY, the probability of combination TTFields being cost‐effective was 0%. In addition, TTFields plus SOC exhibited similar efficacy (1.12 QALYs and 1.14 QALYs) and costs ($309,822 and $312,531) in the treatment of squamous cell carcinoma (SCC) and non‐squamous cell carcinoma (NSCC) populations.

**Conclusions:**

In the United States, TTFields plus SOC as second‐line treatment was not a more cost‐effective strategy for patients with mNSCLC. Of the analyzed regimens, TTFields plus ICI was associated with most significant health benefits.

## Graphical abstract

We evaluated the cost‐effectiveness of TTFields plus SOC versus SOC. TTFields plus ICI has the most advantageous health benefits in mNSCLC. TTFields plus SOC is not a more cost‐effectivenss strategy for mNSCLC in the USA.

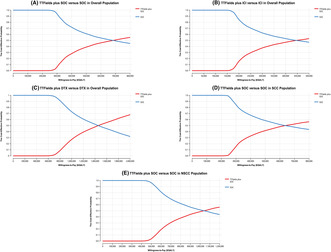

## 1 INTRODUCTION

Lung cancer is the deadliest and second most common form of cancer in the USA, with approximately 238,340 diagnoses and 127,070 deaths forecast in 2023 alone.^1^ Non‐small cell lung cancer (NSCLC) patients comprise 85% of the lung cancer patient population, and roughly 70% of these patients are initially diagnosed only when the disease is locally advances or metastatic, exhibiting a 5‐year overall survival (OS) rate of just 26%.^2^, ^3^ Only one in four NSCLC patients are eligible for surgical resection, and over 60% of these individuals undergo chemotherapeutic treatment.^3^, ^4^ Despite these treatments, affected patients tend to exhibit relatively short OS of just 10–14 months, with over half ultimately developing progressive disease (PD).^5^, ^6^, ^7^, ^8^

Efforts to treat advanced recurrent or metastatic NSCLC (mNSCLC) remain a persistent clinical challenge. Several countries and regulatory agencies have approved immune checkpoint inhibitor (ICI)‐based regimens or docetaxel (DTX) for advanced NSCLC patients that have undergone prior treatment.^9^, ^10^, ^11^ Second‐line ICI or DTX treatment, however, contributes to a median OS of just 13.8 months in individuals with cancer driver genes‐negative.^12^, ^13^, ^14^ Further innovative strategies are thus essential to adequately treat individuals with recurrent or metastatic disease. Tumor‐treating field (TTFields) is a recently developed form of antitumor therapy that relies on the application of alternating electric fields of low frequency and intermediate frequency to interfere with mitotic activity in tumor cells and to modulate the composition of the tumor microenvironment.^15^ TTFields treatment has been shown to trigger stress responses that culminate in immunogenic cell death (ICD), thereby triggering systemic antitumor immunity in a manner that can synergize with immunotherapeutic treatments.^15^, ^16^ The Phase III LUNAR (NCT02973789) trial found that the combination of TTFields and standard of care (SOC) resulted in the improvement of mNSCLC patient OS relative to SOC alone (TTFields plus SOC group: median OS, 13.2 vs. 9.9 months; hazard ratio [HR], 0.74; 95% confidence interval [CI], 0.56–0.98; *p* = 0.035; TTFields plus ICI group: median OS, 18.5 vs. 10.8 months; HR, 0.63; 95% CI, 0.41–0.96; *p* = 0.03; TTFields plus docetaxel [DTX] group: median OS, 11.1 vs. 8.7 months; HR, 0.81; 95% CI, 0.55–1.19; *p* = 0.28).^16^ These survival benefits emphasize the promise of this novel means of treating advanced NSCLC, providing hope to affected patients and suggesting that the combination of local TTFields treatment and systemic therapy may receive approval from appropriate regulatory bodies and enter into widespread clinical practice.

While clinical trials conducted to date have convincingly demonstrated that the TTFields regimen is safe and effective, it is also expensive and only suitable for the treatment of a relatively small subset of NSCLC patients. Given the implications that this novel treatment strategy may have for patients and clinicians, there is a clear need for an economic analysis aimed at establishing the degree to which this therapeutic approach is justifiable from a cost‐effectiveness perspective in an effort to further support the clinical uptake of TTFields treatment. As such, the present study was developed to evaluate the cost‐effectiveness of TTFields plus SOC (ICI and DTX) relative to SOC alone when used as second‐line treatments for mNSCLC patients from a US payers' perspective.

## 2 MATERIALS AND METHODS

The Phase III LUNAR randomized controlled trial (RCT) was the source of all data related to patients, treatment approaches, survival benefits, and safety outcomes. This trial was performed from February 2017 to November 2021, and additional detailed data were obtained from conference abstracts and NovoCure Ltd.^16^ The present study was performed in accordance with the International Society for Pharmacoeconomics and Outcomes Research guidelines for Reporting Standards for CHEERS 2022^17^, ^18^ (Table S1).

### 2.1 Patients and interventions

The hypothetical patient population for this study was comprised of 276 mNSCLC patients (43.5% (*n* = 120) squamous cell carcinoma [SCC] and 56.5% (*n* = 156) non‐SCC [NSCC] patients) with progressive disease (PD) during or after undergoing platinum‐based chemotherapy. Prior ICI treatment of these patients was permitted, and all individuals in this population with an Eastern Cooperative Oncology Group performance status (ECOG PS) ≤2 were randomized into TTFields plus SOC (49.6% [*n* = 137]) and SOC (50.4% [*n* = 139]) treatment groups.^16^ The TTFields plus SOC group was further subdivided as follows: (i) 48.2% of patients underwent TTFields treatment (150 kHz ≥8 h/day) plus ICI (1:1:1 pembrolizumab [200 mg], nivolumab [360 mg], and atezolizumab [1200 mg] every 3 weeks), and (ii) 41.0% of patients underwent TTFields plus DTX (75 mg/m^2^ treatment once every 3 weeks).^9^, ^18^ Chemotherapy drug doses were calculated by assuming all participants to be 64‐year‐old males with a weight, height, and body surface area of 70 kg, 170 cm, and 1.84 m^2^, respectively.^18^, ^19^ For further details regarding these different treatment strategies, see Supplementary Table S2. After undergoing such treatment, 18.0% and 26.0% of the patients in these respective groups developed PD, at which time they were administered the best supportive care (BSC).^16^ Terminal care was provided to all patients that experience cancer‐related mortality.^9^, ^16^

### 2.2 Model construction and overview

A decision‐analytical Markov model was established using TreeAge Pro 2022. This model sought to simulate the process of disease progression by including three mutually exclusive health states (PFS, PD, and death) (Supplementary Figure S1). All patients were in the PFS state at the time of model initiation and had a chance to transition to the PD state or the death state. Patients in the PD state also had a chance to transition to the death state during each cycle. The death state served as the end of the cycle for each patient. Each cycle was 6 weeks in length and the model was run with a 15‐year time horizon, at the end of which almost all patients in both treatment groups were expected to be deceased. Primary study outcomes included total costs, life years (LYs), quality‐adjusted life‐years (QALYs), and incremental cost‐effectiveness ratio (ICER) values. ICERs were calculated based on the incremental costs of a regimen per unit of QALY gained, providing an effective metric for cost‐effectiveness. The willingness‐to‐pay (WTP) threshold selected for this study was $150,000/QALY from a US payers' perspective, as in prior analyses.^19^, ^20^ An annual discount rate of 3% was applied for all costs and health‐related outcomes.^19^

Transition probability values were calculated by using Kaplan–Meier curves to extract data related to short‐term OS and PFS. The Weibull, log‐logistic, log‐normal, exponential, and Gompertz distributions were used for survival curve fitting, and the resultant curves were assessed visually, in light of clinical rationality, and using the Akaike and Bayesian information criterion (AIC and BIC) (Figure S2 and Table S3). Ultimately, the Weibull distribution exhibited the best fit and was selected for use.^19^ These analyses were completed using GetData Graphics Digitizer (v 2.26), R Studio (v 4.2.2), and Matlab (v R2020a). Transition probability values over time were computed with the following formula:

$$(1-\exp\left\{\lambda \left({t-u)}^{\gamma }-\lambda t^{\gamma }\right\}\right)$$

where *u* is the Markov period, *t* is the current model period, and *λ* and *γ* respectively denote the scale and shape parameters (Table 1).^19^

**TABLE 1:** Clinical and health parameters.

Variable	Baseline value (Range)	References	Distribution
Clinical parameters			
Weibull survival model for OS			
TTFields plus SOC in overall population	Scale = 0.059090, Shape = 0.927193	(16)	NA
SOC in overall population	Scale = 0.059479, Shape = 1.031140	(16)	NA
TTFields plus ICI in overall population	Scale = 0.055872, Shape = 0.861250	(16)	NA
ICI in overall population	Scale = 0.050078, Shape = 1.040615	(16)	NA
TTFields plus DTX in overall population	Scale = 0.051529, Shape = 1.096903	(16)	NA
DTX in overall population	Scale = 0.068828, Shape = 1.054559	(16)	NA
TTFields plus SOC in SCC population	Scale = 0.065642, Shape = 0.927734	(16)	NA
SOC in SCC population	Scale = 0.027290, Shape = 1.373620	(16)	NA
TTFields plus SOC in NSCC population	Scale = 0.062240, Shape = 0.937843	(16)	NA
SOC in NSCC population	Scale = 0.096890, Shape = 0.858270	(16)	NA
Weibull survival model for PFS			
TTFields plus SOC in overall population	Scale = 0.185990, Shape = 0.856320	(16)	NA
SOC in overall population	Scale = 0.234310, Shape = 0.805790	(16)	NA
Rate of post‐discontinuation therapy			
TTFields plus SOC	0.180 (0.144–0.216)	(16)	Beta
SOC	0.260 (0.208–0.312)	(16)	Beta
Risk for main AEs in TTFields plus SOC			
Dyspnea	0.070 (0.056–0.084)	(16)	Beta
Anemia	0.080 (0.064–0.096)	(16)	Beta
Pneumonia	0.110 (0.088–0.132)	(16)	Beta
WBC count decreased	0.140 (0.112–0.168)	(16)	Beta
Risk for main AEs in SOC			
Anemia	0.080 (0.064–0.096)	(16)	Beta
Fatigue	0.080 (0.064–0.096)	(16)	Beta
Pneumonia	0.110 (0.088–0.132)	(16)	Beta
WBC count decreased	0.140 (0.112–0.168)	(16)	Beta
Health Parameters			
Utility and disutility			
Utility of PFS	0.650 (0.520–0.780)	(19)	Beta
Utility of PD	0.430 (0.344–0.516)	(19)	Beta
Disutility of anemia	0.073 (0.058–0.088)	(18, 20)	Beta
Disutility of WBC count decreased	0.090 (0.072–0.108)	(18, 20)	Beta
Disutility of pneumonia	0.090 (0.072–0.108)	(20)	Beta
Disutility of fatigue	0.751 (0.601–0.901)	(21)	Beta
Disutility of dyspnea	Unreported	NA	NA
Discount rate	0.03 (0–0.05)	(18)	Uniform

Abbreviations: DTX, docetaxel; ICER, incremental cost‐effectiveness ratio; ICI, immune checkpoint inhibitor; LYs, life‐years; NSCC, non‐squamous cell carcinoma. QALYs, quality‐adjusted life‐years; SCC, squamous cell carcinoma; SOC, standard of care; TTFields, tumor‐treating fields.

### 2.3 Utility and cost inputs

Quality of life (QoL)‐related data were not included in the LUNAR trial, so the respective average utility values applied to PFS and PD were 0.65 and 0.43 based on prior reports^20^ (Table 1). Average utility values were adjusted for the disutility associated with adverse events (AEs) of Grade 3 or higher impacting over 5% of participating patients^19^, ^21^, ^22^ (Table 1). Costs taken into consideration for this study included costs related to treatment, laboratory testing, tumor imaging, drug administration, AE management, BSC, and terminal care (Table 2). The costs of TTFields treatment were obtained from NovoCure Ltd., while the costs of all drugs included in these analyses were from the Centers for Medicare & Medicaid Services.^24^ Other medical costs were computed with the help of previous reports.^19^, ^20^, ^23^, ^25^ All costs were adjusted for inflation to 2023 dollars using the US Consumer Price Index.^26^

**TABLE 2:** Cost estimates.

Parameters	Baseline value (Range)	References	Distribution
Treatment cost, $			
TTFields per month	24,876 (19,901–29,851)	NovoCure Ltd.	Gamma
Nivolumab per cycle	21,929 (17,543–26,315)	(22)	Gamma
Pembrolizumab per cycle	21,924 (17,539–26,309)	(22)	Gamma
Atezolizumab per cycle	19,843 (15,874–23,812)	(22)	Gamma
Docetaxel per cycle	259 (207–311)	(22)	Gamma
Cost of AEs			
TTFields plus SOC	8570 (6856–10,284)	(18, 23)	Gamma
SOC	8479 (6783–10,175)	(18, 23)	Gamma
Administration per cycle	163 (130–196)	(18)	Gamma
Laboratory and tumor imaging per cycle	638 (510–766)	(18)	Gamma
Terminal care per patient	10,850 (8680–13,020)	(19)	Gamma
Best supportive care per cycle	609 (487–731)	(24)	Gamma

Abbreviations: SOC, standard of care; TTFields, tumor‐treating fields.

### 2.4 Sensitivity and scenario analyses

The effects of uncertainty for different variables on the results of this model and its overall robustness were performed through a series of univariate and probabilistic sensitivity analyses. For these analyses, over 20 different key parameters were varied within ±20% of baseline values as in prior studies.^19^, ^20^ Utility values and AE incidence fit a β‐distribution, while costs fit a γ‐distribution.^19^, ^25^

For one‐way sensitivity analyses, a tornado diagram was used to examine the impact of individual variables on ICER values when a single parameter was varied throughout the defined range. For probabilistic sensitivity analyses aimed at assessing simultaneous changes in multiple variables, 10,000 Monte Carlo simulations were performed, and all parameters were randomly sampled from appropriate distributions as recommended based on parameter types.^19^, ^25^ The odds of a given strategy being cost‐effective with different WTP thresholds were assessed by generating cost‐effectiveness acceptability curves and incremental cost‐effectiveness scatter plots.

As the TTFields therapy is very costly, the likelihood of each of the three treatment groups (TTFields plus either SOC, ICI, or DTX) being cost‐effective if the price of TTFields treatment was 100%, 50%, 40%, 30%, 20%, and 10% of current prices when using the same $150,000/QALY WTP threshold.

## 3 RESULTS

### 3.1 Base‐case analysis

In the overall patients, the calculated efficacy (total cost) was 1.23 QALYs ($319,358) in the TTFields plus SOC group and 0.88 QALYs ($106,798) in the SOC group, for an ICER of $613,379/QALY. Similarly, the efficacy (total cost) was 1.58 QALYs ($338,688) in the TTFields plus ICI group and 0.99 QALYs ($112,748) in the ICI group, for an ICER of $387,542/QALY, while the efficacy (total cost) was 0.89 QALYs ($298,477) in the TTFields plus DTX group and 0.74 QALYs ($98,912) in the DTX group, for an ICER of ($1,359,559/QALY) (Table 3). When not taking QoL adjustments or discounting rates into consideration, the life expectancies for patients in the TTFields plus SOC, TTFields plus ICI, and TTFields plus DTX groups were 0.73 LYs (8.76 months), 1.28 LYs (15.36 months), and 0.27 LYs (3.24 months), respectively, relative to the corresponding treatments in the absence of TTFields therapy (Table 3).

**TABLE 3:** Results of the base‐case analysis.

Treatment	Total cost, $	Overall LYs	Overall QALYs	ICER, $	
				per LY	per QALY
Overall population					
SOC	106,798	1.81	0.88	Reference	Reference
TTFields plus SOC	319,358	2.55	1.23	289,858	613,379
ICI	112,748	2.06	0.99	Reference	Reference
TTFields plus ICI	338,688	3.34	1.58	176,377	387,542
DTX	98,912	1.49	0.74	Reference	Reference
TTFields plus DTX	298,477	1.76	0.89	741,091	1,359,559
Squamous cell carcinoma population					
SOC	105,448	1.52	0.76	Reference	Reference
TTFields plus SOC	309,822	2.30	1.12	263,302	566,912
Non‐squamous cell carcinoma population					
SOC	104,176	1.92	0.93	Reference	Reference
TTFields plus SOC	312,531	2.34	1.14	496,915	976,969

Abbreviations: LYs, life‐years; QALYs, quality‐adjusted life‐years; ICER, incremental cost‐effectiveness ratio; TTFields, tumor‐treating fields; SOC, standard of care; ICI, immune checkpoint inhibitor; DTX, docetaxel; SCC, squamous cell carcinoma; NSCC, non‐squamous cell carcinoma.

For the SCC patient cohort, the efficacy (total cost) was 1.12 QALYs ($309,822) in the TTFields plus SOC group and 0.76 QALYs ($105,448) in the SOC group for an ICER of $566,912/QALY, whereas in the NSCC cohort, the efficacy (total cost) was 1.14 QALYs ($312,531) and 0.93 QALYs ($104,176) in these two respective groups, for an ICER of $976,969/QALY (Table 3). These results clearly demonstrated that TTFields plus SOC is not a cost‐effective option to treat mNSCLC patients, irrespective of whether ICI or DTX was used for the SOC regimen (Table 2).

### 3.2 Sensitivity analyses

**FIGURE 1:**
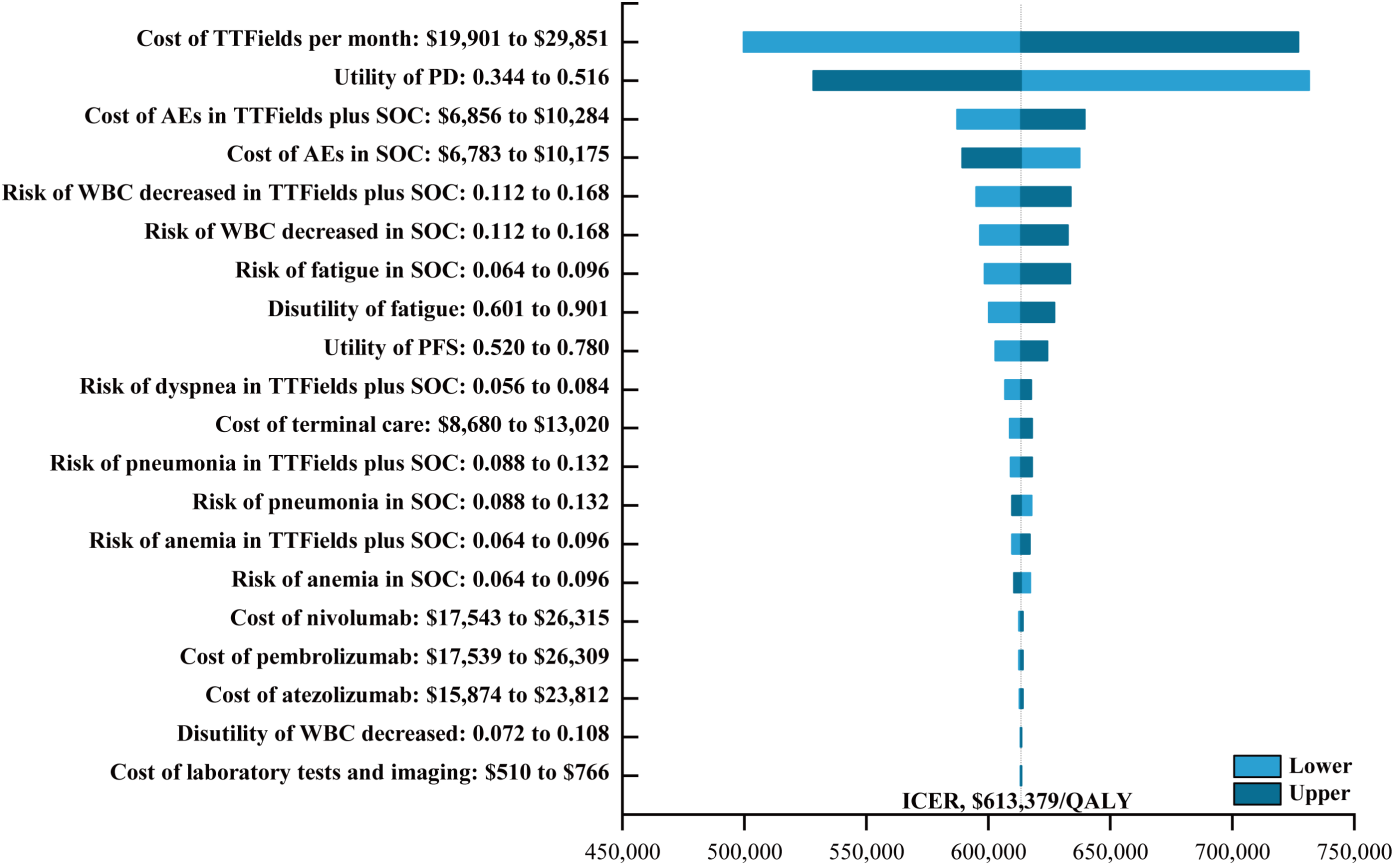
The one‐way sensitivity analyses for the TTFields plus SOC compared to the SOC. AEs, adverse events; ICER, incremental cost‐effectiveness ratio; PD, progressive disease; PFS, progression‐free survival; QALY, quality‐adjusted life‐year; SOC, the standard of care; TTFields, tumor‐treating fields; WBC, white blood cell.

In one‐way sensitivity analyses, the variables that had the greatest impact on model robustness included the cost of TTFields treatment, PD utility values, and AE‐related costs in the TTFields plus SOC and SOC groups. TTFields costs ranged from $19,901 to $29,851, resulting in proportional shifts in ICER values. When varying the tested parameters within 20% above or below baseline, ICER values ranged from $499,655/QALY to $727,103/QALY, demonstrating that TTFields plus SOC is a less cost‐effective approach than SOC alone when employed at the designated WTP threshold of $150,000/QALY (Figure 1). None of the tested parameters significantly altered the main conclusions of this study, thus highlighting the robust nature of the established model.

**FIGURE 2:**
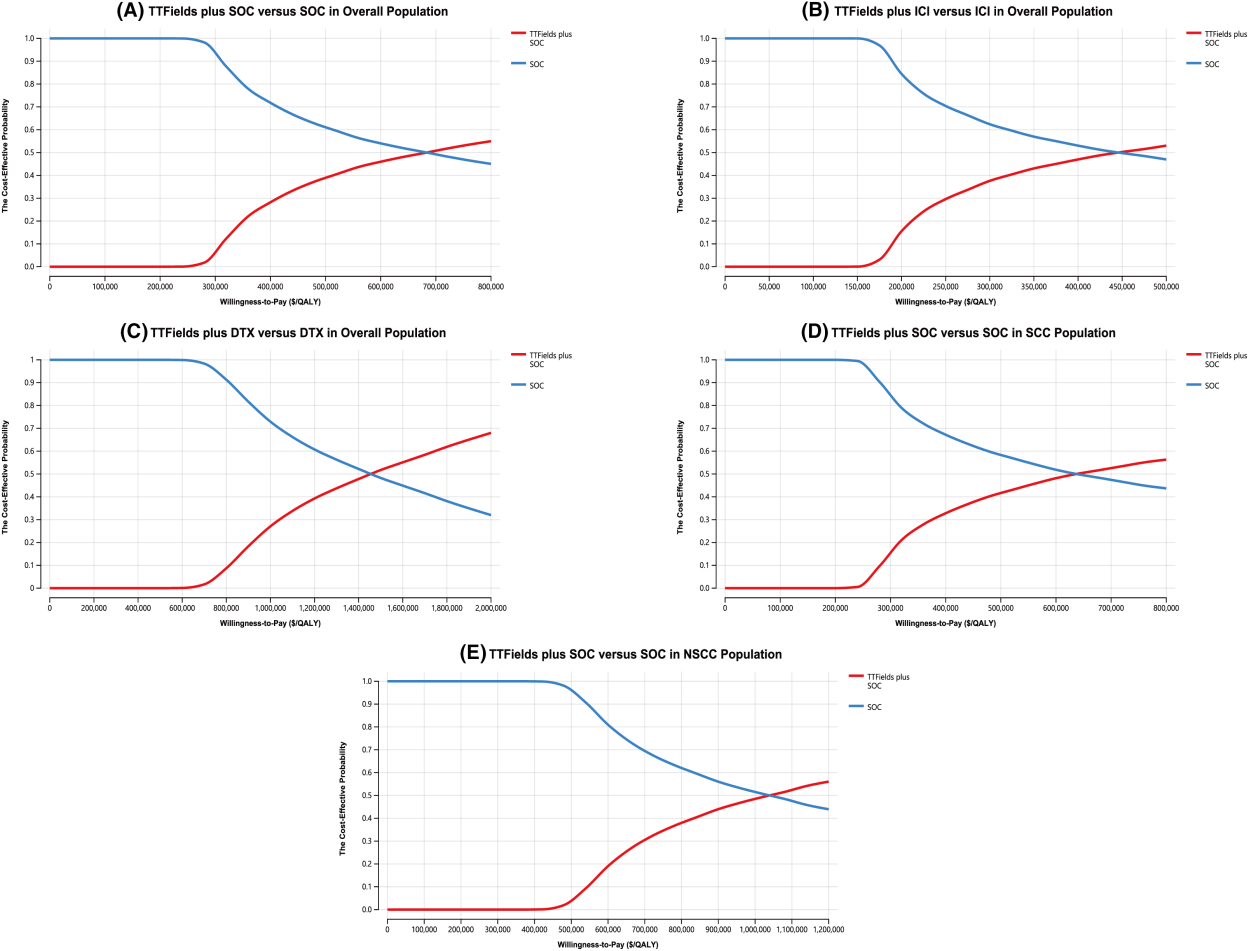
The cost‐effectiveness acceptability curves for the TTFields plus SOC compared to the SOC in overall population (A), the TTFields plus ICI compared to the ICI in overall population (B), the TTFields plus DTX compared to the DTX in overall population (C), the TTFields plus SOC compared to the SOC in SCC population (D), and the TTFields plus SOC compared to the SOC in NSCC population (E). DTX, docetaxel; ICI, immune checkpoint inhibitor; NSCC, non‐squamous cell carcinoma; QALY, quality‐adjusted life‐year; SCC, squamous cell carcinoma; SOC, the standard of care; TTFields, tumor‐treating fields.

Probabilistic sensitivity analysis results were presented using acceptability curves, which revealed that TTFields combination regimens were more likely to be cost‐effective as the WTP threshold rose. For the overall mNSCLC patient cohort, the TTFields plus SOC, TTFields plus ICI, and TTFields plus DTX regimens exhibited a 50% chance of being cost‐effective at respective WTP thresholds of $685,000/QALY, $490,000/QALY, and $1,450,000/QALY (Figure 2). For patients with SCC and NSCC, TTFields plus SOC exhibited a 50% chance of being cost‐effective at respective $640,000/QALY and $1,035,500/QALY WTP thresholds (Figure 2). There was a 0% chance of TTFields combination regimens being cost‐effective when using the defined WTP threshold of $150,000/QALY to represent a US payers' perspective (Figure S3).

### 3.3 Scenario analyses

When reducing the cost of TTFields treatment by 90% and 70%, the ICER values fell to $101,621/QALY and $141,546/QALY for the TTFields plus SOC versus SOC and TTFields plus ICI versus ICI comparisons, such that these approaches were cost‐effective at the $150,000/QALY WTP threshold. TTFields plus DTX group was found to be unlikely to be cost‐effective irrespective of the degree of cost reduction (Supplementary Materials Table S4).

## 4 DISCUSSION

In patients with driver oncogene‐negative NSCLC that progress following first‐line treatment, combination chemotherapy or ICI monotherapy can be employed for second‐line treatment.^9^, ^10^ This relatively simplified treatment scheme, however, warrants further diversification, spurring a growing number of clinical studies evaluating a range of combination treatment regimens, many of which have failed to provide meaningful benefits to patients.^27^, ^28^ In the recent LUNAR study, TTFields plus SOC outperformed SOC alone when used to treat individuals with mNSCLC.^16^ Cancer patients inevitably face particularly high medical expenses and associated financial burdens as a consequence of high treatment costs and medical resource limitations. In the United States, lung cancer‐related medical expenditures were estimated to range from $15.19 to 18.84 billion in 2020 alone, and are forecast to rise with additional increases in cancer incidence and patient mortality.^29^ In light of rising health care costs, there is an ever‐growing need for cost‐based oncology, prompting the present analysis of the cost‐effectiveness of TTFields plus SOC as a second‐line treatment option for mNSCLC patients.

No prior reports to our knowledge have compared the cost‐effectiveness of TTFields plus SOC (ICI or DTX) treatment for mNSCLC patients to that of SOC (ICI or DTX) alone. In the base‐case analysis, the ICER values for the TTFields plus SOC, TTFields plus ICI, and TTFields plus DTX regimens were $613,379/QALY, $387,542/QALY, and $1,359,559/QALY, respectively, relative to the corresponding SOC, ICI, and DTX regimens. As all of these values were well above the WTP threshold, this clearly emphasized the fact that TTFields plus SOC regimens are not a cost‐effective treatment option in the USA at present. One‐way sensitivity analyses revealed that the factors that most significantly influenced these ICER values were the cost of TTFields and the PD utility value, with treatment costs having a particularly pronounced effect on these ICERs. Additional probabilistic sensitivity analyses were performed aimed at exploring how likely TTFields plus SOC regimens were to be cost‐effective if prices were reduced. While the odds of TTFields combination regimens being cost‐effective at the $150,000/QALY WTP threshold, these odds did improve as the price of TTFields decreased for the TTFields plus SOC or TTFields plus ICI regimens, although there was no change in the cost‐effectiveness of TTFields plus DTX treatment. As the high costs associated with novel anticancer treatments can impose a severe economic burden on patients and society as a whole through rising Medicare costs, economic instability, bankruptcy, and a need for less efficacious treatments that contribute to a greater risk of death,^30^, ^31^ efforts to lower TTFields treatment costs represent a promising and realistic approach to enhancing the cost‐effectiveness of this regimen. These results thus offer a valuable source of guidance for future clinical applications and guideline approval.

To date, there have been insufficient economic analyses focused on TTFields‐based combination regimens for NSCLC patients. However, three studies have drawn similar conclusions when evaluating TTFields as a treatment for other forms of cancer.^32^, ^33^, ^34^ Those studies were conducted based on data derived from the EF‐14 trial (NCT00916409).^35^, ^36^ Guzauskas et al., Connock et al., and Waschke et al. respectively computed that the ICER values for TTFields plus maintenance temozolomide as compared to temozolomide alone in newly‐diagnosed glioblastoma patients were $197,336/QALY (WTP, $100,000/QALY), €510,273/QALY (WTP, €100,000/QALY), and €351,909/QALY (WTP, €100,000/QALY), from the perspectives of the American, French, and German health care systems.^32^, ^33^, ^34^ These results found that the poor cost‐effectiveness of TTFields combination therapy was primarily attributable to treatment costs, the utility of PD, and variations in WTP thresholds among countries. Relative to ICI treatment alone, TTFields plus ICI yielded significant improvements in mNSCLC patient QLAYs, with a trend toward a greater degree of cost‐effectiveness. In contrast, TTFields plus DTX did not yield any significant improvement in QALYs while also costing significantly more such that there was no opportunity for this regimen to be a cost‐effective option. Economic analyses have proposed US WTP thresholds ranging from $100,000/QALY and $300,000/QALY,^37^, ^38^ and the use of the highest of these values resulted in an increase in the odds of TTFields plus ICI being cost‐effective from 0% to 37%. The high costs associated with the TTFields‐based treatment of mNSCLC represent a major potential burden for patients, health care systems, and society such that these results can inform mNSCLC patient treatment planning and health insurance policymaking in the Unites States.

This analysis offers several important advantages. For one, this is the first study to our knowledge to use up‐to‐date evidence when assessing the relative benefits of TTFields plus SOC and SOC alone in mNSCLC patients. While TTFields has offered some promise as a means of treating certain types of solid tumors, from a cost‐effectiveness perspective, the feasibility of this regimen remains limited. Second, the benefits of the TTFields plus SOC regimen were compared to SOC alone from a US payers' perspective, providing results tailored to the American health care system that can provide valuable support for clinicians, policymakers, and individuals in the health care finance sector seeking to make decisions regarding particular regimens. These results can also provide a foundation for multilateral TTFields price negotiations in domestic and international settings. Third, this economic analysis included subgroup analyses of patients with specific pathological types of NSCLC. While TTFields plus SOC was more efficacious when treating SCC patients relative to NSCC patients, the economic outcomes were comparable for both patient groups, extending the survival of SCC and NSCC patients by 0.78 LYs (9.36 months) and 0.42 LYs (5.04), respectively, relative to SOC alone such that both subgroups exhibited ICER values above the WTP threshold for US payers. This aligns with prior evidence that SCC and NSCC are distinct from one another with respect to immune microenvironment composition and other characteristics, with SCC patients tending to exhibit better survival outcomes.^39^, ^40^, ^41^, ^42^ Analyzing economic models specific to particular NSCLC patient populations may provide more robust guidance for treatment‐related planning.

This study is also subject to some limitations. For one, the established Markov model compared TTFields plus SOC and SOC based on the results of a Phase III trial with a relatively short follow‐up period, potentially contributing to underestimates of efficacy. However, the survival curves were extracted with a relatively stable Weibull distribution model, allowing for a more reliable assessment of patient survival benefits. This model also did not take costs related to immune‐related AEs, low‐grade AEs, and adverse device events into consideration. Sensitivity analysis results were not impacted by modulating AE‐associated variables within the defined range. Third, the treatment‐related costs used in this study were derived from the US Medicare database, while other costs were from published reports. Given the lack of any study‐specific data pertaining to QoL, utility data were obtained from published studies pertaining to patients with advanced or metastatic NSCLC, and univariate sensitivity analyses suggested that these utility values did not significantly impact overall model stability. The LUNAR trial also failed to provide PFS curves for patients that underwent TTFields plus ICI/DTX treatment for the overall patient population, or for patients that underwent TTFields plus SOC treatment for the NSCC and SCC patient subgroups. The PFS benefits for these groups were consistent with the benefits of TTFields plus SOC in the overall population, per the approach published by Ding et al., emphasizing a need for caution when interpreting these study results.^19^

## 5 CONCLUSION

This study is the first economic evaluation focused on long‐term survival outcomes for NSCLC patients undergoing TTFields plus SOC treatment from a US payers' perspective. These results offer evidence supporting future clinical decision‐making regarding the suitability of TTFields combination treatment regimens for the management of NSCLC patients in the USA when taking economic factors and health insurance policies into consideration. The present results suggest that TTFields plus SOC is unlikely to be a cost‐effective option for the overall patient population. The TTFields plus ICI regimen was associated with the most substantial health benefits. These results may provide guidance for mNSCLC patient treatment planning and for the design of health insurance policies in the United States and other nations. Future changes in treatment policies or Medicare coverage may render this a cost‐effective therapeutic option.

## AUTHOR CONTRIBUTIONS

**Kun Liu:** Conceptualization (lead); formal analysis (lead); methodology (lead); software (lead); validation (lead); visualization (lead); writing – original draft (lead); writing – review and editing (lead). **Youwen Zhu:** Conceptualization (lead); formal analysis (lead); investigation (equal); methodology (equal); software (equal); validation (lead); visualization (lead); writing – original draft (lead); writing – review and editing (lead). **Hong Zhu:** Conceptualization (lead); funding acquisition (equal); methodology (lead); project administration (equal); resources (equal); software (equal); supervision (equal); validation (equal); visualization (equal); writing – original draft (equal); writing – review and editing (equal). **Manting Zeng:** Conceptualization (lead); formal analysis (lead); funding acquisition (lead); investigation (equal); project administration (lead); resources (lead); supervision (lead); validation (equal); visualization (equal); writing – original draft (equal); writing – review and editing (equal).

## FUNDING INFORMATION

This work was partly supported by the Clinical Research Project of Xiangya Hospital (grant number, 2016L06 to H.Z.).

## CONFLICT OF INTEREST STATEMENT

All of the authors have indicated that they have no competing interests in the content of the article.

## ETHICS STATEMENT

This article is based on previously conducted studies and does not contain any new studies with human participants or animals performed by any of the authors, it does not require the approval of the independent ethics committee.

## Supporting information


Figure S1.
Figure S2.
Figure S3.
Table S1.
Table S2.
Table S3.
Table S4.

## Data Availability

All authors had full access to all of the data in this study and take complete responsibility for the integrity of the data and accuracy of the data analysis. The datasets generated and/or analyzed during the current study are available from the corresponding author upon reasonable request.
